# The prognostic value of neurofilament levels in patients with sepsis-associated encephalopathy – A prospective, pilot observational study

**DOI:** 10.1371/journal.pone.0211184

**Published:** 2019-01-24

**Authors:** Johannes Ehler, Axel Petzold, Matthias Wittstock, Stephan Kolbaske, Martin Gloger, Jörg Henschel, Amanda Heslegrave, Henrik Zetterberg, Michael P. Lunn, Paulus S. Rommer, Annette Grossmann, Tarek Sharshar, Georg Richter, Gabriele Nöldge-Schomburg, Martin Sauer

**Affiliations:** 1 Department of Anesthesiology and Intensive Care Medicine, University Medical Center Rostock, Rostock, Germany; 2 Department of Neuroimmunology, Institute of Neurology, University College London, London, United Kingdom; 3 Moorfields Eye Hospital, The National Hospital for Neurology and Neurosurgery, London, United Kingdom; 4 Department of Neurology, University Medical Center Rostock, Rostock, Germany; 5 Department of Internal Medicine, Intensive Care Unit, University Medical Center Rostock, Rostock, Germany; 6 Department of Molecular Neuroscience, Institute of Neurology, University College London, London, United Kingdom; 7 UK Dementia Research Institute at University College London, London, United Kingdom; 8 Department of Psychiatry and Neurochemistry, Institute of Neuroscience and Physiology, the Sahlgrenska Academy at the University of Gothenburg, Mölndal, Sweden; 9 Clinical Neurochemistry Laboratory, Sahlgrenska University Hospital, Mölndal, Sweden; 10 Department of Neurology, Medical University Vienna, Vienna, Austria; 11 Institute for Diagnostic and Interventional Radiology, University Medical Center Rostock, Rostock, Germany; 12 Department of Neuro-anesthesiology and Intensive Care Medicine, Saint-Anne Teaching Hospital, Paris-Decartes University, Paris, France; 13 Laboratory of Human Histopathology and Animal Models, Institut Pasteur, Paris, France; Azienda Ospedaliero Universitaria Careggi, ITALY

## Abstract

Sepsis-associated encephalopathy (SAE) contributes to mortality and neurocognitive impairment of sepsis patients. Neurofilament (Nf) light (NfL) and heavy (NfH) chain levels as biomarkers for neuroaxonal injury were not evaluated in cerebrospinal fluid (CSF) and plasma of patients with sepsis-associated encephalopathy (SAE) before. We conducted a prospective, pilot observational study including 20 patients with septic shock and five patients without sepsis serving as controls. The assessment of SAE comprised a neuropsychiatric examination, electroencephalography (EEG), magnetic resonance imaging (MRI) and delirium screening methods including the confusion assessment method for the ICU (CAM-ICU) and the intensive care delirium screening checklist (ICDSC). CSF Nf measurements in sepsis patients and longitudinal plasma Nf measurements in all participants were performed on days 1, 3 and 7 after study inclusion. Plasma NfL levels increased in sepsis patients over time (p = 0.0063) and remained stable in patients without sepsis. Plasma NfL values were significantly higher in patients with SAE (p = 0.011), significantly correlated with the severity of SAE represented by ICDSC values (R = 0.534, p = 0.022) and correlated with a poorer functional outcome after 100 days (R = -0.535, p = 0.0003). High levels of CSF Nf were measured in SAE patients. CSF NfL levels were higher in non-survivors (p = 0.012) compared with survivors and correlated with days until death (R = -0.932, p<0.0001) and functional outcome after 100 days (R = -0.749, p<0.0001). The present study showed for the first time that Nf levels provide complementary prognostic information in SAE patients indicating a higher chance of death and poorer functional/cognitive outcome in survivors.

## Introduction

During the last decades the main focus of sepsis care has been directed towards short- and long-term survival of patients [1]. Consequently patient management has improved reducing the overall mortality [2]. An important contributor to mortality and long-term morbidity is sepsis-associated encephalopathy (SAE) [3–6]. SAE is defined as a diffuse brain dysfunction secondary to sepsis and without evidence of a primary CNS infection or encephalopathy due to other reasons [7]. The pathophysiology of SAE is still unexplained but risk factors are emerging [8–11]. Structural evidence for brain injury in sepsis comes from imaging and neuroanatomy studies [9–13]. Clinical assessment of SAE is hampered by the altered level of consciousness due to sedation and the need for mechanical ventilation [6,14]. Neuropsychiatric examination, electroencephalography (EEG), neuroimaging and laboratory tests permit to monitor SAE [15–17]. Diagnostic accuracy especially of clinical examination and EEG monitoring, however remain low in more severe cases potentially confounded by the use of sedation [17]. Furthermore, the need for prolonged registration of EEG to detect abnormalities over time is not practicable in the ICU setting and previous studies showed no association between EEG and brain dysfunction detected by CAM-ICU [18]. In this context body fluid biomarkers may be of diagnostic value [6,19,20]. A common limitation to previous studies on SAE was that biomarkers investigated are not specific for the neuro-axonal compartment and results have been contradictory [21–26]. A more specific biomarker for neuro-axonal injury, the neurofilament proteins (Nf) can be accurately measured from the cerebrospinal fluid (CSF) and blood and consistently correlated with brain injury, disease severity and survival in a range of neurological diseases [9,27–32]. Nf proteins are an important part of the axonal cytoskeleton and represent an architectonic stable tube system [30]. They are classified as intermediate filaments of type IV [30]. As a consequence of axonal injury Nf are released into the extracellular fluid and can be measured by ELISA technique [30]. This is the first study on the value of neurofilament heavy (NfH) and neurofilament light chains (NfL) in cerebrospinal fluid (CSF) and plasma of patients with SAE. The aim is to evaluate the potential suitability of Nf as biomarkers to detect SAE, septic brain injury and to predict outcome in patients with sepsis.

## Methods

### Study design and ethical protocol

We conducted a prospective, longitudinal single-center exploratory study at three ICU at the university medical center Rostock, Germany. The patient recruitment period was between May 2012 and November 2016. All patients or their legal representatives signed a written informed consent form before study inclusion. The study was registered as a clinical trial (ClinicalTrials.gov: NCT02442986) and was approved by the local ethics board at Rostock University (A 2012–0058).

Inclusion criteria for participants were patients with an age ≥ 18 years and an inclusion within 24 hours after the beginning of severe sepsis or septic shock according to the sepsis criteria used at that time [33]. Exclusion criteria for all participants were evidence for any preexisting neuromuscular disease like diabetic, alcoholic polyneuropathy or inflammatory neuropathies. Additionally, patients with a history of CNS diseases like dementia, ischemia or hemorrhage were excluded. Furthermore, coagulopathy with active bleeding, no informed consent by legal representatives, high-dose glucocorticosteroid treatment, preexisting renal replacement therapy and expected death within 12 hours were exclusion criteria.

Participants with an expected length of ICU stay of more than 48 hours but without sepsis and without brain dysfunction were included as controls. Except for MRI and lumbar puncture these control subjects had the same longitudinal assessment as sepsis patients.

### Multimodal assessment protocol for sepsis-associated encephalopathy

#### Clinical assessment and long-term follow-up

Patients were longitudinally assessed for their time of ICU and hospital stay by an interdisciplinary team consisting of experienced neurologists and intensivists. Recommended severity of disease scales including the Acute Physiology and Chronic Health Evaluation II (APACHE-II) score at ICU admission and the Sepsis-related Organ Failure Assessment (SOFA) score at study days 1, 3, 7 and 28 were used [34–36]. All ICU patients were treated as recommended by international guidelines [2,33]. The length of ICU and hospital stay, days on the ventilator and 28- and 100-day survival were recorded from all participants. The Barthel index (BI) before hospital admittance and at day 100 after study inclusion was used to assess patients’ activities of daily living and to evaluate patients’ long-term functional outcome [37,38]. A standardized telephone interview with the patients or their legal representatives was conducted to ascertain the BI at day 100.

#### Neuropsychiatric assessment

SAE was defined as a diffuse brain dysfunction secondary to sepsis and without evidence of a primary CNS infection or encephalopathy due to other reasons [7].

All participants were assessed for clinical signs of brain dysfunction by neuropsychiatric examination within one day after study inclusion by an experienced neurologist (MW). This included a detailed medical history from the patient or their legal representatives for early clinical signs of brain dysfunction like confusion, agitation or reduced level of consciousness [39–41]. Furthermore, the evaluation of EEG recordings and the evaluation of CSF results completed the neuropsychiatric assessment performed by the neurologist. A standardized neurologic examination included brainstem function. Based on this neuropsychiatric assessment the diagnosis of SAE was made.

#### Confusion assessment method for the ICU (CAM-ICU) and Intensive Care Delirium Screening Checklists (ICDSC)

The patients’ level of consciousness was assessed by physicians experienced in intensive (MS, JE) and neuro-intensive care (JE) on day 1, 3, 7 and 28 using the Glasgow Coma Scale (GCS) and the Richmond Agitation and Sedation Scale (RASS) [36,42]. The longitudinal assessment of brain dysfunction was performed using CAM-ICU and ICDSC as validated scales to detect signs of delirium [9,39–41]. According to recommendation CAM-ICU was only performed in patients with a RASS above -4. A patient was defined as CAM-ICU positive if CAM-ICU screening was positive at least at one time point of assessment.

#### Electroencephalography and magnetic resonance imaging

Within 72 hours after study inclusion all patients underwent EEG examinations. EEG recordings (ED 14; Madaus Schwarzer, Munich, Germany) were performed over a time of 30 mins and were assessed by an experienced neurologist (MW). Details on the methods of EEG recording and the classification of EEG findings using the Young scale were described elsewhere [9,43]. Furthermore, a standardized MRI protocol was used in septic patients to detect brain injury in SAE as described in detail before [9,13]. In brief, septic shock patients were examined by MRI (1.5-T magnet system MAGNETOM Avanto, Siemens Healthcare, Erlangen, Germany; 3.0-T mangnet system MAGNETOM Verio, Siemens Healthcare) as soon they were stable for in house transfer. The extent of white matter hyperintensities (WMH), an imaging marker of septic brain damage, was assessed by a neuroradiologist (AG) according to a validated scale [13]. This 4-graded scale describes WMH in the septic brain according to their number and size. The scale comprises grade 0 (no lesions), grade 1 (punctiform lesions), grade 2 (patchy or confluent lesions) and grade 3 (diffuse lesions) [13].

### Neurofilament proteins

CSF samples for Nf measurements were derived from patients with clinical evidence for SAE and were taken by lumbar puncture within 72 hours after study inclusion. This time frame was set to achieve a hemodynamic stabilization of septic shock patients before lumbar puncture. Plasma samples were taken at days 1, 3 and 7. Neurofilaments were measured using two validated in-house developed ELISA kits [32,44,45]. All samples were batch analyzed in duplicates. The mean intra-assay coefficient of variation in our study was 3.24%.

### Statistical analysis

All statistical analyses were performed in SAS (version 9.4). Normality was tested graphically and using Shapiro–Wilk statistics. Gaussian data were compared using the T-Test and non–Gaussian data with the non-parametric Wilcoxon test. A two–way unbalanced ANOVA (general linear model, GLM) was used for comparing more than two independent variables. Weighted power calculations were performed for an alpha of 0.5. Correlation analyses were performed using Pearson’s R for Gaussian and Spearman’s R for non–Gaussian data. Multiple correlations were corrected by the Bonferroni method.

## Results

### Patient demographics

**Fig 1:**
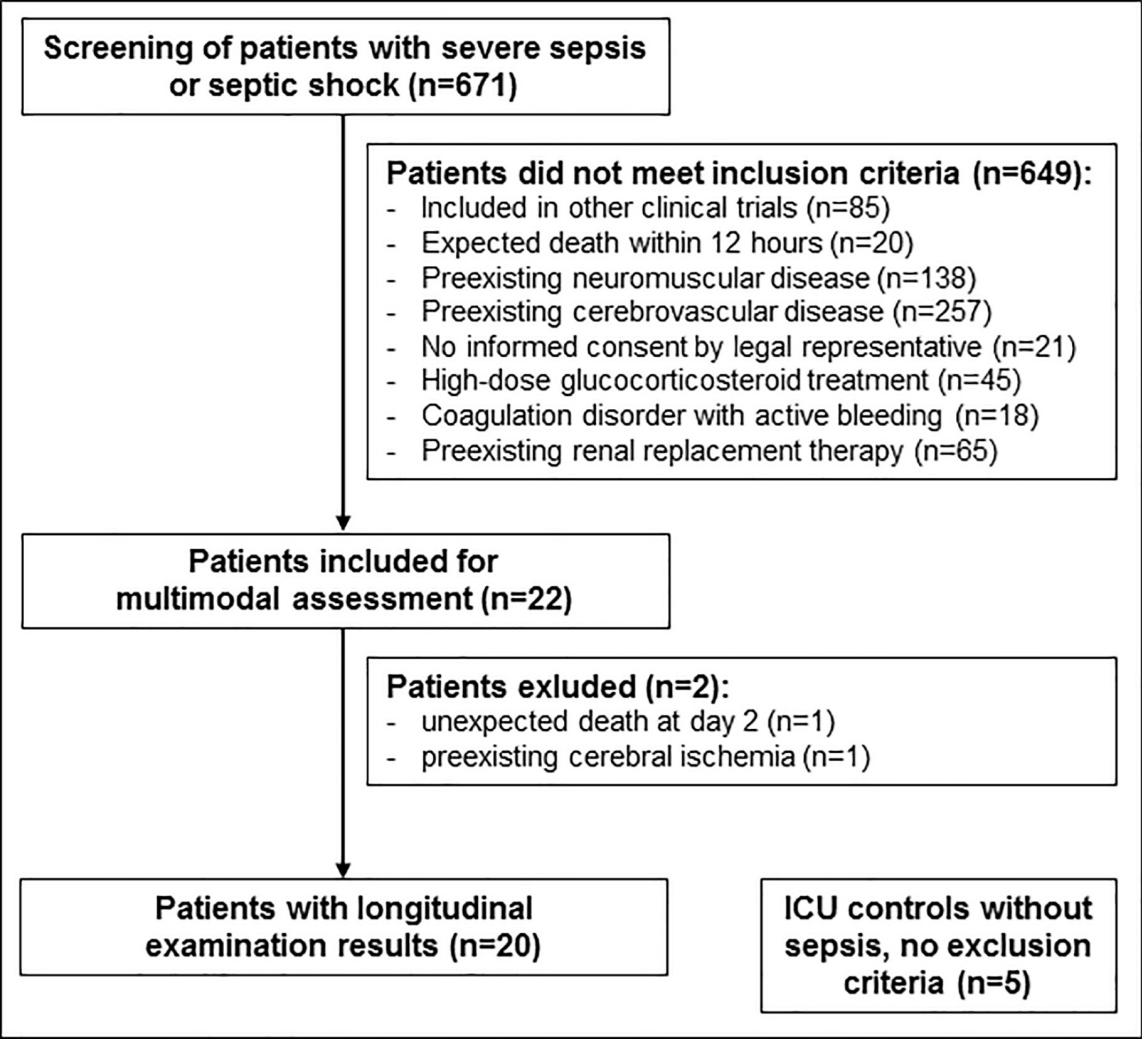
Study flow chart showing the prospective patient enrollment. ICU Intensive Care Unit.

Twenty five critically ill ICU patients were prospectively included, 20 patients with sepsis and five patients without sepsis serving as controls (Fig 1). MRI reports of 13 included patients were previously published with a focus on neuroaxonal injury in sepsis (9). In the present study a total of 20 patients with severe sepsis or septic shock, mean (SD) age 66.7 (14.0) years, eight male and twelve female, and five matched ICU patients without sepsis, mean (SD) age 61.2 (24.7) years, three of them male and two female, were enrolled (Table 1).

**Table 1:** Patient characteristics of 25 study participants.

Patient	ICU cohort	Age/ Gender	BI before ICU	Sepsis condition	Sepsis focus	APACHE-II/ Worst SOFA	Ventilation (days)
1	Sepsis	63/F	100	Shock	Abdomen	20/18	72
2	Sepsis	82/F	90	Shock	Urogenital	29/15	12
3	Sepsis	85/F	95	Severe Sepsis	Abdomen	14/16	0
4	Sepsis	73/M	100	Shock	Abdomen	42/15	20
5	Sepsis	57/M	100	Shock	Abdomen	27/11	2
6	Sepsis	55/M	100	Shock	Abdomen	12/6	0
7	Sepsis	80/F	95	Shock	Urogenital	24/12	2
8	Sepsis	64/M	100	Shock	Soft tissue	11/14	27
9	Sepsis	72/F	35	Shock	Urogenital	9/4	0
10	Sepsis	44/M	95	Shock	Abdomen	40/8	10
11	Sepsis	76/F	100	Shock	Pulmo	39/12	16
12	Sepsis	74/F	90	Shock	Pulmo	23/13	9
13	Sepsis	72/F	100	Shock	Urogenital	38/10	0
14	Sepsis	75/M	100	Severe Sepsis	Urogenital	37/10	2
15	Sepsis	79/F	100	Shock	Soft tissue	22/6	1
16	Sepsis	32/M	100	Shock	Abdomen	19/9	4
17	Sepsis	54/F	100	Shock	Soft tissue	48/11	8
18	Sepsis	55/F	75	Shock	Soft tissue	39/14	6
19	Sepsis	60/F	70	Shock	Soft tissue	23/12	12
20	Sepsis	81/M	100	Shock	Urogenital	38/12	20
21	Control	74/M	100	None	n.a.	23/3	1
22	Control	63/M	100	None	n.a.	9/5	0
23	Control	18/F	100	None	n.a.	14/5	1
24	Control	74/M	100	SIRS	n.a.	17/5	0
25	Control	77/F	100	SIRS	n.a.	22/7	1

*APACHE-II* Acute physiology and chronic health evaluation score, *BI* Barthel index (activities of daily living), *F* Female, *ICU* Intensive care unit, *M* Male, *n*.*a*. Not applicable, *SIRS* Systemic inflammatory response syndrome, *SOFA* Sepsis-related organ failure assessment score.

The mean BI of the controls (100) was not significantly different from the mean (SD) BI of the sepsis cohort (92.3 (15.9), p>0.05).

### Clinical assessment of sepsis-associated encephalopathy in sepsis and control patients

SAE was diagnosed in 18 of 20 sepsis patients by neuropsychiatric assessment. CAM-ICU screening was positive for brain dysfunction in 16 of 20 participants. None of the five control subjects showed clinical signs of brain dysfunction according to neuropsychiatric assessment, CAM-ICU or ICDSC screening. Sepsis patients presented significantly higher mean (SD) ICDSC values in comparison to the control group (ICDSC 3.3 (2.2) in sepsis vs. 0.8 (0.45) in controls, p = 0.025).

### Electroencephalography and magnetic resonance imaging in sepsis and control patients

EEG examination was performed in 24 of 25 patients. EEG was not available in one patient due to technical problems. The grade of EEG abnormalities differed between sepsis and control patients (Table 2). None of the sepsis patients showed normal EEG activity (Table 2). In contrast, the majority of control patients showed normal alpha activity. Unexpectedly, triphasic waves were detected in one young female control patient (case #23) without evidence for SAE or any CNS disease. The patient refused cranial MRI examination which prevented further clarification.

**Table 2:** Electroencephalography and magnetic resonance imaging results from 25 study participants.

Patient cohort		EEG findings				MRI findings	
	Normal activity	Theta waves	Delta waves	Triphasic waves	Burst-suppression pattern	WMH present	Ischemic lesions present
**SAE**^**a**^	0/18	10/18	8/18	0/18	0/18	9/11	3/11
**No SAE**	1/2	1/2	0/2	0/2	0/2	0/2	0/2
**Control**^**b**^	3/4	0/4	0/4	1/4	0/4	n.a.	n.a.

*EEG* Electroencephalography, *MRI* Magnetic resonance imaging, *n*.*a*. Not applicable, *WMH* White matter hyperintensities, *SAE* Sepsis-associated encephalopathy.

^a^ MRI reports available from 11/18 SAE patients

^b^ EEG reports available from 4/5 controls.

WMH were detected in nine out of 13 sepsis patients with available MRI examinations. Additionally, subacute ischemic lesions were detected in three sepsis patients respectively. All EEG and MRI data are summarized in Table 2.

### Long-term outcome after 100 days

Seven of 25 patients died within 100 days after study inclusion. Six non-survivors belonged to the sepsis group and one patient to the control group. The mean (SD) BI of sepsis survivors was lower than the mean (SD) BI of survivors of the control group (78.21 (29.7 vs. 95.0 (10.0), p>0.05).

### Plasma neurofilament levels in sepsis and control patients

**Fig 2:**
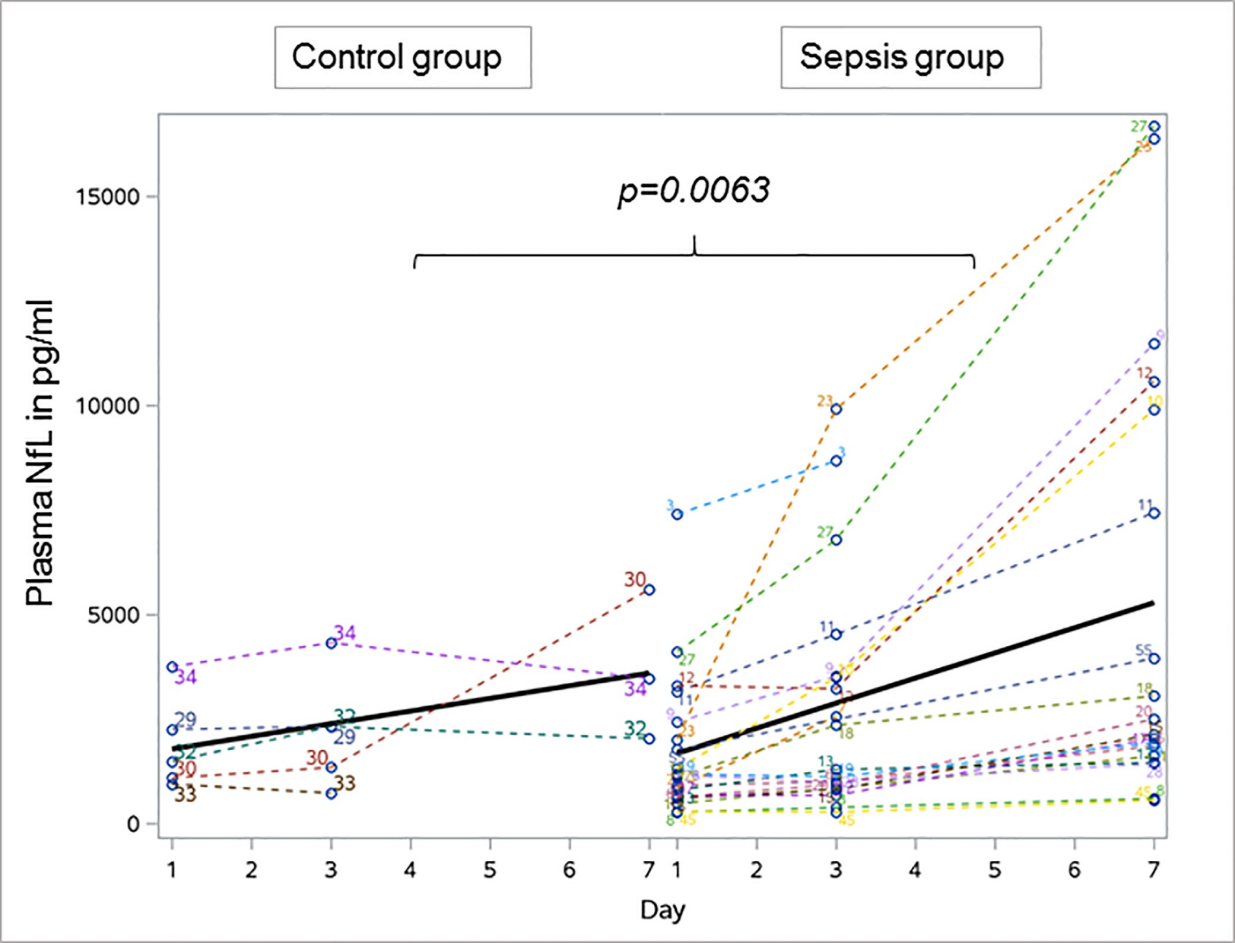
Longitudinal profile of neurofilament light chains over time in 20 sepsis and five control patients. The NfL levels significantly increased in sepsis patients over time and remained stable in controls. Bold line indicates the development of mean plasma neurofilament levels over time. NfL Neurofilament light chains.

Plasma NfL and NfH values were compared between 20 sepsis and five control patients. Significant differences were present for NfL when comparing sepsis and control patients over time. The mean NfL values at study day 1 were not statistically different between the groups,but over time NfL plasma values of sepsis patients were significantly higher in comparison to controls (GLM, p = 0.0063, Fig 2). Within the sepsis group plasma NfL levels significantly increased from day 1, mean (SD) NfL 1723.4 (1711.5) pg/mL to day 7, mean (SD) 5309.6 (5373.9) pg/mL (p<0.001) which was not observed in the control group (day 1, mean (SD) NfL 1905.2 (1151.9) pg/mL vs. day 7, mean (SD) NfL 3701.3 (1794.8) pg/mL, p>0.05).

Plasma NfH values were not significantly different between sepsis and control patients at day 1. Within the sepsis group a significant increase was observed from day 1, mean (SD) NfH 17.6 (41.5) ng/mL to day 7, mean (SD) NfH 163.4 (596.0) ng/mL (p = 0.043). This difference was not present in the control group (day 1, mean (SD) NfH 100.3 (221.4) ng/mL vs. day 7, mean (SD) NfH 519.9 (666.9) ng/mL, p>0.05).

An overview about the different development of NfL and NfH levels over time is given in Table 3.

**Table 3:** Plasma neurofilament levels in sepsis patients and controls.

**Neurofilament Light (NfL)**	**Sepsis group** **Mean (SD), pg/mL**	**Patients, No. (%)** **(n = 20)**	**Control group** **Mean (SD), pg/mL**	**Patients, No (%)** **(n = 5)**	***p Value*^*a*^**
Day 1	1723.4 (1711.5)	20 (100)	1905.2 (1151.9)	5 (100)	p>0.05
Day 3	2753.1 (2774.5)	20 (100)	2208.0 (1363.5)	5 (100)	p>0.05
Day 7	5309.6 (5373.9)	18 (90)	3701.3 (1794.8)	3 (60)	p>0.05
***p value day 1 vs*. *day 7***^***b***^	p<0.001		p>0.05		
**Neurofilament Heavy (NfH)**	Sepsis group Mean (SD), ng/mL	Patients, No. (%) (n = 20)	Control group Mean (SD), ng/mL	Patients, No (%) (n = 5)	***p Value***^***a***^
Day 1	17.6 (41.5)	20 (100)	100.3 (221.4)	5 (100)	p>0.05
Day 3	18.9 (63.2)	20 (100)	163.1 (350.2)	5 (100)	p>0.05
Day 7	164.3 (596.0)	18 (90)	519.9 (666.9)	3 (60)	p = 0.016
***p value day 1 vs*. *day 7***^***b***^	p = 0.043		p>0.05		

No, Number; SD, Standard deviation.

^a^ p values calculated by comparing sepsis patients and controls.

^b^ p values calculated by comparing neurofilament levels day 1 vs. day 7 within each study group.

Power calculations on these data indicate that a group size of n = 134 for plasma NfL, n = 126 for plasma NfH is needed to reach a power of 80% for separating sepsis from controls.

### Plasma neurofilament levels in patients with sepsis-associated encephalopathy

**Fig 3:**
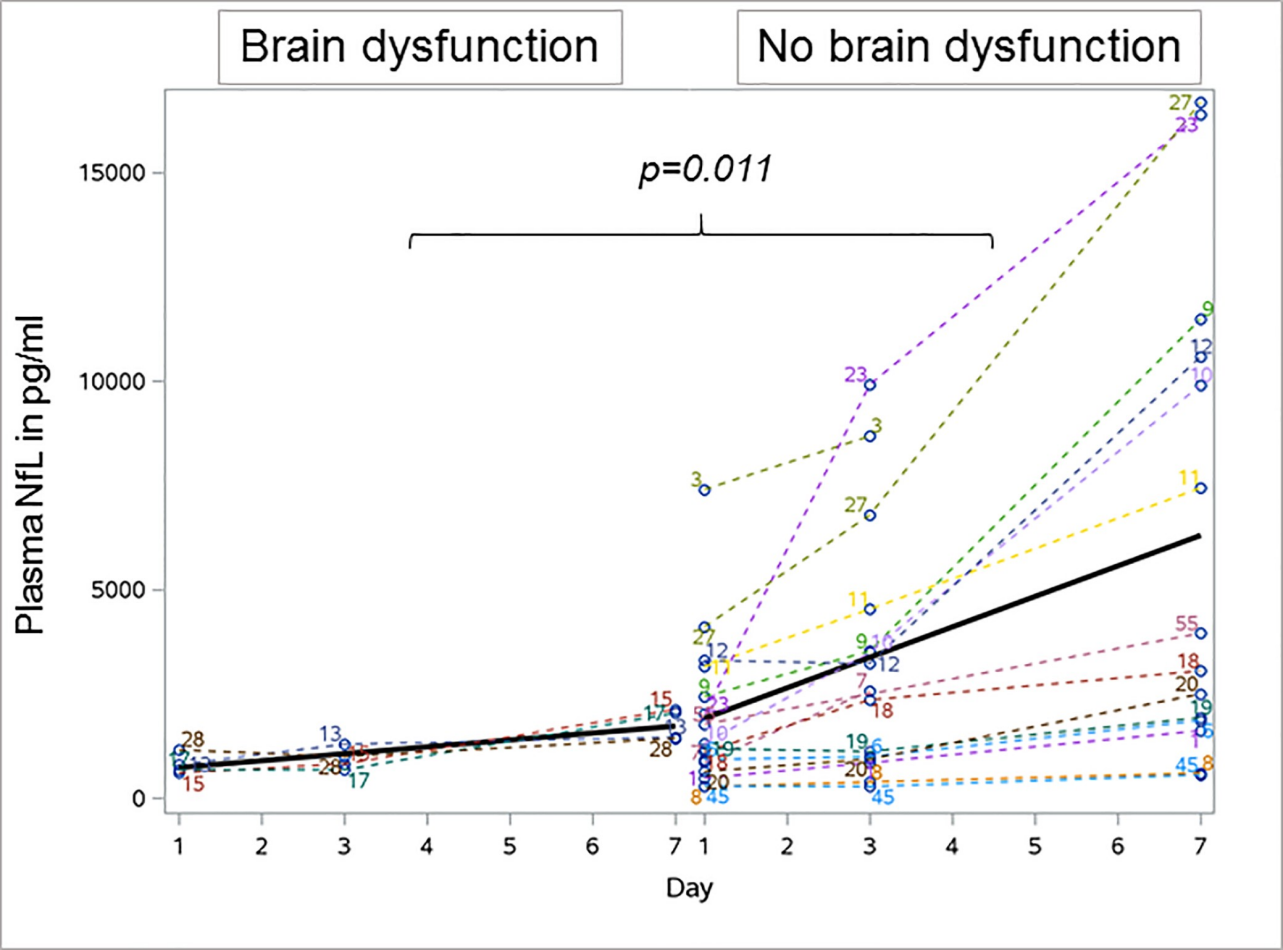
Longitudinal profile of plasma neurofilament light chain levels in 16 sepsis patients with brain dysfunction and four patients without brain dysfunction. NfL levels significantly increased in patients with brain dysfunction over time which was not observed in patients without brain dysfunction. Bold line indicates the development of mean plasma neurofilament levels over time. NfL Neurofilament light.

Nf levels of 16 CAM-ICU positive patients were compared to four CAM-ICU negative patients. Mean (SD) NfL levels in CAM-ICU negative patients increased from 808.8 (245.2) pg/ml at day 1 to 1762.8 (370.5) pg/ml at day 7. Mean (SD) NfL levels in CAM-ICU positive patients significantly increased from 1952.0 (1849.2) pg/ml at day 1 to 6323.0 (5723.3) pg/ml at day 7 (p = 0.001). This increase over time was significantly stronger in CAM-ICU positive compared to CAM-ICU negative patients (GLM, p = 0.011, Fig 3). Next we corrected for missing samples using mixed models which confirmed the above finding (p = 0.0007). Peak concentrations of plasma NfL correlated with higher ICDSC values in sepsis patients (R = 0.534, p = 0.022). Mean (SD) NfH levels in CAM-ICU positive patients were higher at study day 1 (NfH 22.0 (45.6) ng/ml) compared to CAM-ICU negative patients (mean NfH 0.0 ng/ml) and further increased in CAM-ICU positive patients to a mean value of 211.3 (673.7) ng/ml at day 7, which was not observed in CAM-ICU negative patients (mean NfH 0.0 ng/ml). No significant group difference was observed for the development of NfH levels over time (Table 4).

**Table 4:** Plasma neurofilament levels in patients with and without brain dysfunction detect by the Confusion assessment method for the ICU.

**Neurofilament Light (NfL)**	**Brain dysfunction** **Mean (SD), pg/mL**	**Patients, No. (%)** **(n = 16)**	**No brain dysfunction** **Mean (SD), pg/mL**	**Patients, No (%)** **(n = 4)**	***p Value***^***a***^
Day 1	1952.0 (1849.2)	16 (100)	808.8 (245.2)	4 (100)	p>0.05
Day 3	3205.0 (2940.7)	16 (100)	945.5 (265.3)	4 (100)	p>0.05
Day 7	6323.0 (5723.3)	14 (87.5)	1762.8 (370.5)	4 (100)	p>0.05
***p value day 1 vs*. *day 7***^***b***^	p = 0.001		p>0.05		
**Neurofilament Heavy (NfH)**	**Brain dysfunction** **Mean (SD), ng/mL**	**Patients, No. (%)** **(n = 16)**	**No brain dysfunction** **Mean (SD), ng/mL**	**Patients, No (%)** **(n = 4)**	***p Value***^***a***^
Day 1	22.0 (45.6)	16 (100)	0	4 (100)	p>0.05
Day 3	23.7 (70.3)	16 (100)	0	4 (100)	p>0.05
Day 7	211.3 (673.7)	14 (87.5)	0	4 (100)	p>0.05
***p value day 1 vs*. *day 7***^***b***^	p = 0.043		p>0.05		

No, Number; SD, Standard deviation.

^a^ p values calculated by comparing sepsis patients and controls.

^b^ p values calculated by comparing neurofilament levels day 1 vs. day 7 within each study group.

Power calculations on these data indicate that for plasma NfL a group size of n = 10 is required on day one and of n = 14 on day seven to to reach a power of 80% for separating CAM-ICU positive from CAM-ICU negative patients.

**Fig 4:**
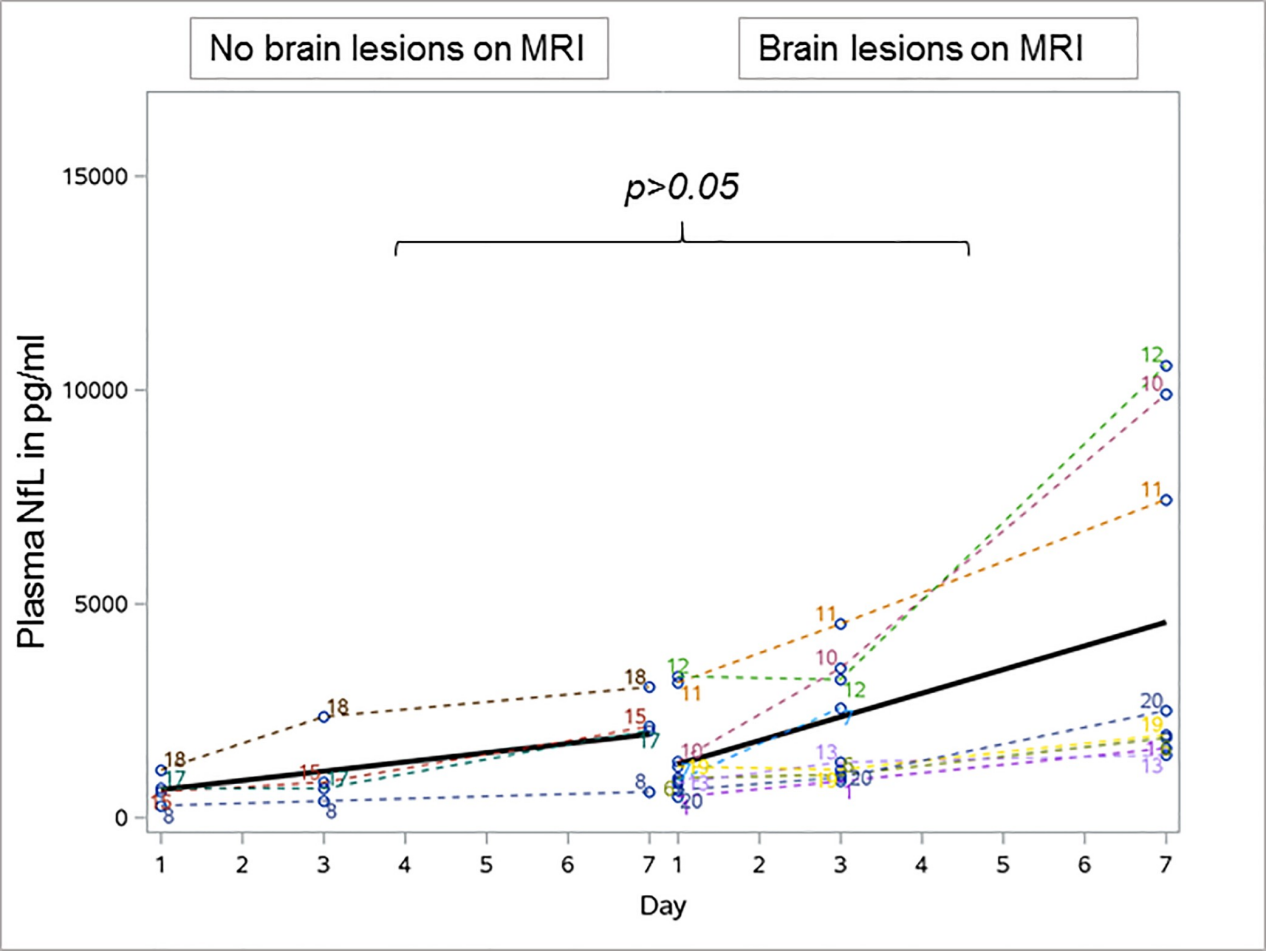
Development of plasma neurofilament light chain levels in patients with no brain lesions and brain lesions seen on magnetic resonance imaging in sepsis. No difference in the plasma NfL increase over time between septic patients with and without brain lesions seen on MRI. Bold line indicates the development of mean plasma neurofilament levels over time. NfL Neurofilament light chain, MRI Magnetic resonance imaging.

MRI results were available from 13 sepsis patients. Septic brain injury, represented by different extents of WMH was detected in nine and not detected in four patients. Further details on MRI results are provided elsewhere (9). Patients with evidence for WMH tended to have higher plasma NfL values (mean (SD) NfL levels at day 1: 1405.0 (1063.5) pg/ml; at day 3: 2110.1 (1373.7) pg/ml; day 7: 4658.9 (3959.0) pg/ml) compared to patients without WMH (mean (SD) NfL at day 1: 665.8 (343.6) pg/ml, p>0.05; day 3: 1058.0 (882.1) pg/ml, p = 0.045; day 7: 1953.3 (1011.4) pg/ml, p>0.05) which correlated with the extent of lesions on MRI (Fig 4). In comparison to patients without WMH a significant increase of plasma NfL levels was detected in patients with WMH between day 1 and day 7 (p = 0.012).

Mean (SD) NfH levels slightly increased in patients with WMH from 22.9 (53.8) ng/ml at day 1 to 355.3 (887.0) ng/ml at day 7 (p>0.05) which was completely different to patients without WMH who did not show an increase of NfH levels over time (NfH 0 ng/ml at all three time points of measurement).

#### Plasma neurofilament levels, 28-day and 100-day mortality in sepsis

Plasma Nf levels of four non-survivors at day 28 were compared to 16 survivors. Mean (SD) NfL levels in non-survivors increased from 2880.5 (3048.4) pg/ml at day 1 to 6724.7 (8367.0) pg/ml at day 7 (p>0.05). In survivors mean (SD) NfL levels at day 1 increased from 1434.1 (1185.6) pg/ml to 5026.6 (4954.9) pg/ml (p = 0.001).

Mean (SD) NfH levels in non-survivors at day 1 increased from 62.0 (77.6) ng/ml to 847.8 (1461.8) ng/ml (p>0.05) compared to survivors with a mean (SD) NfH level of 6.5 (18.0) ng/ml at day 1 and an increase to 27.6 (78.6) ng/ml (p>0.05).

Six non-survivors at day 100 were compared to 14 survivors. Mean (SD) NfL levels of the non-survivors significantly increased from 2765.3 (2417.0) pg/ml at day 1 to 6940.6 (6370.6) pg/ml at day 7 (p = 0.043). In survivors mean (SD) NfL levels increased from 1276.8 (1148.3) pg/ml at day 1 to 4682.3 (5084.2) pg/ml at day 7 (p = 0.001).

Mean (SD) NfH levels of non-survivors increased from 58.7 (60.5) ng/ml at day 1 to 586.2 (1096.2) ng/ml at day 7 (p>0.05). In survivors mean NfH levels increased from 0 ng/ml at day 1 to 2.1 (7.5) ng/ml at day 7 (p>0.05).

#### Highly elevated cerebrospinal fluid neurofilament levels in patients with sepsis-associated encephalopathy

CSF samples were available from 12 of 20 sepsis patients. The mean (SD) time to CSF examination was 2.75 (2.1) days. Mean (SD) levels of NfH were 561.41 (1697.5) ng/ml and 21891.5 (49917.2) pg/ml for NfL (Table 5). In 8 of 20 patients lumbar puncture could not be performed due to disclaimer from legal representative after study inclusion (n = 4), unsuccessful puncture related to patient specific anatomical reasons (n = 3) and contraindication due to local soft tissue infection (n = 1).

**Table 5:** Neurofilament levels in cerebrospinal fluid of twelve sepsis patients.

Patient/ Study code	Study days to LP	Cell count in Mpt/l (Ref <5 Mpt/l)	Protein in mg/l (Ref 150–450 mg/l)	CSF NfH in ng/ml	CSF NfL in pg/ml
1	8	1	324	65.0	4908
2	2	1	561	87.2	9425
4	3	1	353	0	2909
6	2	3	302	25.2	2166
7	2	3	245	85.5	9822
9	2	1	209	53.9	4864
10	3	2	453	71.0	3007
11	1	3	326	79.9	20704
14	1	2	373	51.7	5961
15	3	3	282	177.5	14965
18	2	1	151	5949.9	179432
19	5	1	232	89.8	4535

*LP* Lumbar puncture, *Mpt/l* Megaparticels/liter, *NfH* Neurofilament heavy chain, *NfL* Neurofilament light chain, *Ref* Reference range.

#### Correlation of cerebrospinal fluid neurofilament levels with sepsis-associated encephalopathy, 28-day and 100-day mortality

**Fig 5:**
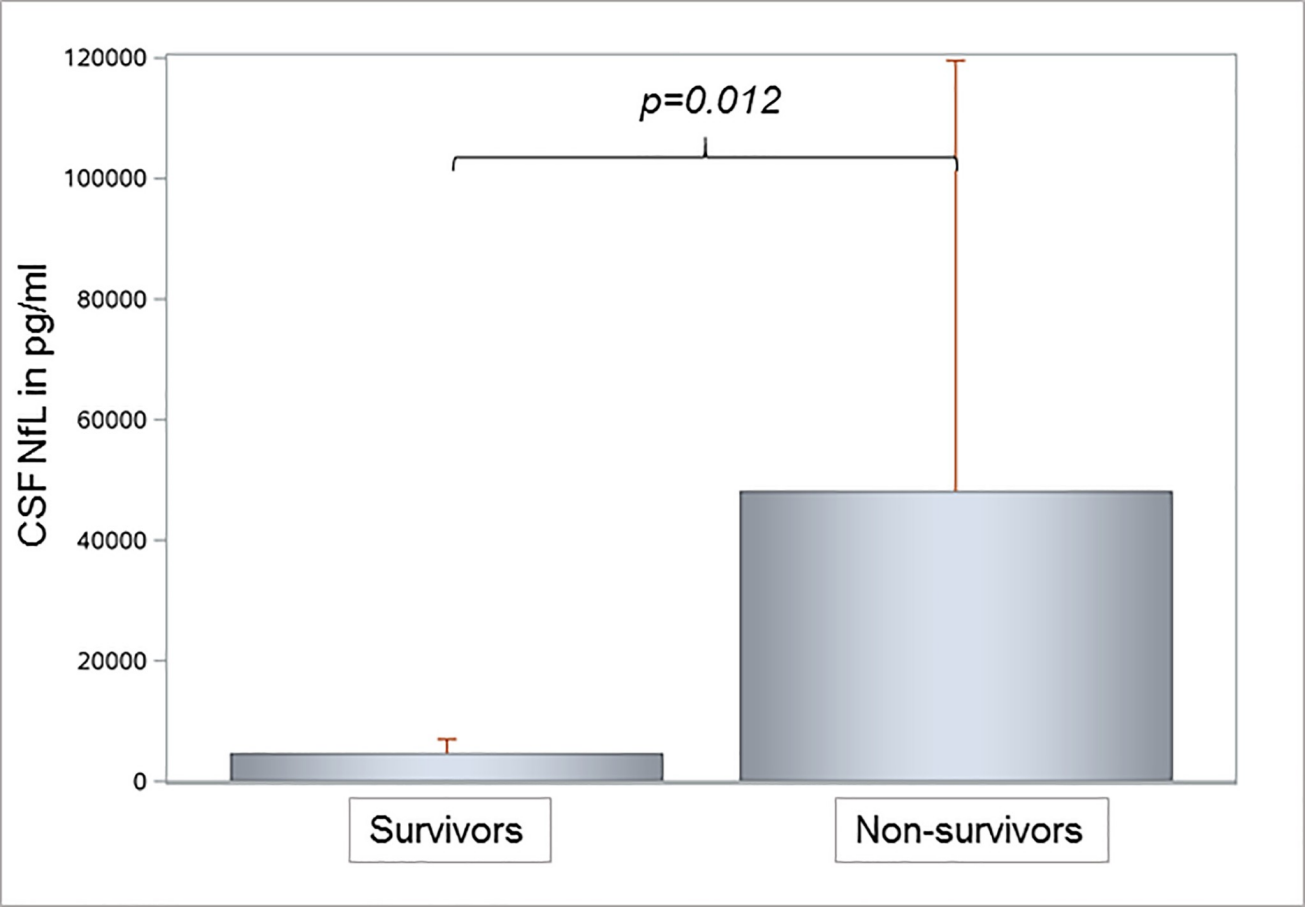
Cerebrospinal fluid neurofilament light chain levels in seven survivors and five non-survivors of sepsis. Significantly higher NFL levels were observed in non-survivors compared to survivors of sepsis. NfL Neurofilament light chain.

Neuropsychiatric examination diagnosed SAE at the beginning of sepsis in all twelve patients with CSF analysis. Therefore, we were not able to compare CSF Nf levels between SAE positive and negative patients. CSF NfL levels were significantly higher in both 28- and 100-day non-survivors. In three non-survivors at day 28 the mean (SD) NfL level of 69,986 (94,939.1) pg/ml was significantly higher compared to a mean (SD) NfL level of 5860 (4027.5) pg/ml of nine survivors (p = 0.021). We measured significantly higher CSF NfL levels in five non-survivors at day 100 with a mean (SD) NfL level of 45,966.2 (74,840.8) pg/ml compared to seven survivors with a mean (SD) NfL level of 4695.3 (2465.3) pg/ml (p = 0.012, Fig 5). CSF NfH levels tended to be higher in septic non-survivors at day 28 (mean (SD) NfH level 2038.4 (3387.5) ng/ml) compared to survivors (mean (SD) NfH level of 69.0 (49.7) ng/ml; p>0.05). This trend was confirmed in non-survivors at day 100 with a mean (SD) NfH level of 1271.6 (2615.7) ng/ml compared to survivors with a mean (SD) NfH level of 54.1 (32.7) ng/ml (p>0.05).

Power calculations on these data indicate that a group size of n = 53 for CSF NfL, n = 72 for CSF NfH is needed to reach a power of 80% for separating survivors from non-survivors.

#### Correlation of cerebrospinal neurofilament levels with brain pathology seen on magnetic resonance imaging

Seven patients with WMH seen on MRI showed significantly higher CSF NfL levels, mean (SD) 8217.3 (6139.6) pg/ml, compared to two patients without WMH, mean (SD) CSF NfL 2958.0 (69.3) pg/ml (p = 0.017). No significant difference in CSF NfH levels was observed between patients with WMH (mean (SD) CSF NfH level 69.2 (23.8) ng/ml) and without WMH seen on MRI (mean (SD) CSF NfH level 35.5 (50.2) ng/ml, p>0.05).

#### Correlation of cerebrospinal fluid and plasma neurofilament levels with long-term functional outcome

We observed a negative correlation between CSF NfH levels and BI before sepsis. A lower BI before hospital admittance was associated with higher CSF NfH levels (R = -0.490, p = 0.0028), which was neither present for CSF NfL nor for both plasma Nf levels. Higher CSF NfL (R = -0.749, p<0.0001) and higher plasma NfL levels (R = -0.535, p = 0.0003) correlated with a lower BI at day 100 representing a poorer clinical outcome of these patients. Additionally, a link to patient outcome was observed by correlating CSF NfH and NfL as well as plasma NfH values with the time to death in non-survivors. Higher Nf levels were associated with shorter survival of patients (Table 6).

**Table 6:** Spearmen’s correlation analysis for cerebrospinal fluid and plasma NfH and NfL values.

Parameter	Cerebrospinal fluid		Plasma	
	NfH	NfL	NfH	NfL
**BI before sepsis**	R = -0.490 **p = 0.0028**	p>0.05	p>0.05	p>0.05
**BI at day 100**	p>0.05	R = -0.749 **p<0.0001**	p>0.05	R = -0.535 **p = 0.0003**
**Days on ICU**	p>0.05	p>0.05	p>0.05	p>0.05
**Days in hospital**	R = 0.571 **p = 0.007**	p>0.05	p>0.05	p>0.05
**Days on ventilator**	p>0.05	p>0.05	p>0.05	p>0.05
**Days until death of non-survivors**	R = -0.657 **p = 0.011**	R = -0.932 **p<0.0001**	R = -0.658 **p = 0.011**	p>0.05

*BI* Barthel index, *ICU* Intensive care unit, *NfH* Neurofilament heavy chains, *NfL* Neurofilament light chains, *n*.*s*. not significant, *SOFA* Sepsis-related organ failure assessment.

## Discussion

This prospective longitudinal exploratory study was conducted to evaluate the prognostic value of Nf levels in patients with SAE. Nf levels are known to be increased in several disorders with neuropsychiatric symptoms [27,31,46]. The potential value for SAE has not yet been investigated. Preexisting studies on SAE analyzed non-specific biomarkers like interleukin-6, neuron-specific enolase (NSE) or S100B protein [19, 21–23,47,48,49]. The results were found to be controversial [19,48,49]. Our group is first to analyze the prognostic value of CSF and plasma NfH and NfL levels in SAE patients which might have importance for the prediction of long-term neurological sequelae and survival in sepsis. Biomarker research on SAE is necessary as most septic shock patients are sedated and mechanically ventilated and are not easily assessed for clinical signs of SAE [6,16,43]. Diagnostic measures to evaluate the extent of septic brain injury, as cerebral MRI or diffusion tensor imaging (DTI), are not universally available and require resources [50,51]. Specific markers of neuroaxonal injury in SAE could help to monitor SAE and to predict neurological outcome.

The highly elevated Nf levels in patients with SAE impressively underline the occurrence of brain injury in sepsis patients [9,10,13,52,53]. As we performed lumbar puncture early during the course of sepsis a close temporal relationship between the occurrence of septic shock and septic brain injury has to be suspected. The extent of brain injury was confirmed by MRI during study follow-up, which was significantly correlated to elevated NfL levels. Both, MRI and Nf results support an early start of sepsis treatment and a rapid hemodynamic stabilization of septic shock patients to prevent CNS and multiple organ failure. This is strikingly obvious with a look at the correlation of CSF Nf levels with time to death in non-survivors. Additionally CSF and plasma NfL levels significantly correlated with BI at day 100. Higher NfL values were associated with a poorer long-term functional outcome of survivors, which underlines the relevance of SAE and the prognostic value of NfL levels. We did not find a correlation between BI before sepsis and BI at day 100 after sepsis which might have been seen otherwise as a confounding factor.

Some limitations of the present study have to be mentioned. The number of study participants was low, which was a consequence of our strict exclusion criteria (a combination of peripheral and central nervous system diseases) for this single-center study. As SAE is a diagnosis of exclusion, we did not include patients with a preexisting neurological disease to prevent a main inclusion bias and to be able to evaluate the development of Nf levels in SAE over time as precise as possible. All patients finally included were otherwise examined by multimodal diagnostics. Our standard MRI examinations might still have underestimated the extent of brain injury of SAE patients. DTI, which was not available for our investigation would have been more accurate to visualize neuroaxonal injury [50].

The temporal relationship between onset and progression of sepsis and development of brain injury is supported by the longitudinal profile of the plasma Nf levels presented here. No differences of Nf levels were measured at study day 1 between sepsis and control patients, which is in agreement with earlier studies showing that NfL is a slow marker reaching its maximum 10–14 days following traumatic brain injury [54]. This is also important as the comparable basal Nf levels were not primarily different between both groups, which could have been a confounder. Over time, the NfL levels increased in SAE patients, particularly so in patients with a positive CAM-ICU and severe SAE as indicated by ICDSC. This is relevant as plasma Nf might act as biomarkers to detect and monitor SAE in septic shock patients. NfL levels in CSF and by tendency in plasma were significantly higher in patients with brain injury seen on MRI. Recently our group demonstrated evidence for two distinct patterns of neuroaxonal injury in sepsis with ischemia and diffuse axonal injury as relevant pathomechanisms [9]. The results of the present study support the diagnostic role of Nf measurements to detect brain injury in sepsis and might support their suitability as potential biomarkers of neuroaxonal injury in SAE patients. This is supported by previous immunohistochemistry findings from human septic brain tissue [9]. We reported on the disruption of white matter axons in post-mortem brains of sepsis patients indicated by staining for nonphosphorylated NfH chains [9]. Immunohistochemistry gave clear evidence for axonal degeneration in sepsis which supports the diagnostic role of Nf measurements in SAE patients.

## Conclusion

This is the first study on the relevance of neurofilament heavy (NfH) and neurofilament light chains (NfL) in cerebrospinal fluid (CSF) and plasma to detect SAE and to predict outcome in patients with sepsis. This prospective, longitudinal, registered study showed that NfL and NfH levels were found to be highly elevated in plasma and CSF of patients with SAE. Nf levels in sepsis correlated with the clinical appearance of SAE, the extent of neuroaxonal injury seen on MRI and with survival. Power calculations indicate that future studies on prediction of sepsis survival will require larger sample sizes compared to studies focused on cognitive/functional outcome in survivors. Given the difficulty in obtaining CSF samples in septic shock patients, the modest gain for study size calculation and the methodological developments we suggest future studies to focus on longitudinal plasma NfL and NfH levels using fourth generation immunoassays.
