# Correction: The prognostic value of neurofilament levels in patients with sepsis-associated encephalopathy - A prospective, pilot observational study

**DOI:** 10.1371/journal.pone.0212830

**Published:** 2019-02-20

**Authors:** Johannes Ehler, Axel Petzold, Matthias Wittstock, Stephan Kolbaske, Martin Gloger, Jörg Henschel, Amanda Heslegrave, Henrik Zetterberg, Michael P. Lunn, Paulus S. Rommer, Annette Grossmann, Tarek Sharshar, Georg Richter, Gabriele Nöldge-Schomburg, Martin Sauer

In Fig 3, the headings above the graph are incorrectly swapped. The left heading should be “No Brain Dysfunction” and the right heading should be “Brain Dysfunction.” The authors have provided a corrected version here.

**Fig 3:**
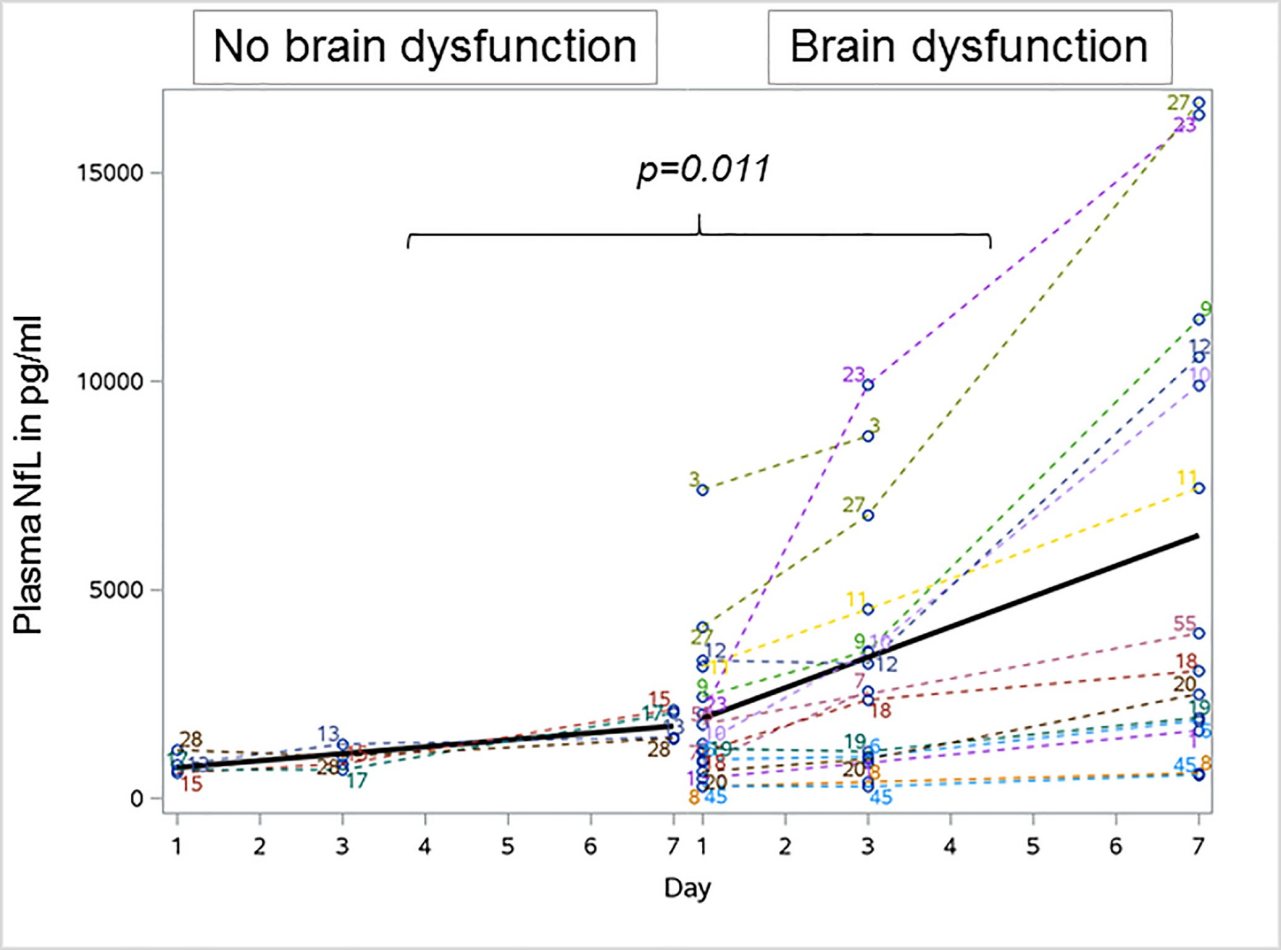
Longitudinal profile of plasma neurofilament light chain levels in 16 sepsis patients with brain dysfunction and four patients without brain dysfunction. NfL levels significantly increased in patients with brain dysfunction over time which was not observed in patients without brain dysfunction. Bold line indicates the development of mean plasma neurofilament levels over time. NfL Neurofilament light.

## References

[1] Ehler J, Petzold A, Wittstock M, Kolbaske S, Gloger M, Henschel J, et al (2019) The prognostic value of neurofilament levels in patients with sepsis-associated encephalopathy–A prospective, pilot observational study. PLoS ONE 14(1): e0211184 10.1371/journal.pone.0211184 30677080 PMC6345472

